# Yersiniabactin is a quorum-sensing autoinducer and siderophore in uropathogenic *Escherichia coli*

**DOI:** 10.1128/mbio.00277-23

**Published:** 2024-01-18

**Authors:** James R. Heffernan, John A. Wildenthal, Hung Tran, George L. Katumba, William H. McCoy, Jeffrey P. Henderson

**Affiliations:** 1 Center for Women’s Infectious Disease Research, Washington University School of Medicine, St. Louis, Missouri, USA; 2 Division of Infectious Diseases, Washington University School of Medicine, St. Louis, Missouri, USA; 3 Department of Internal Medicine, Washington University School of Medicine, St. Louis, Missouri, USA; 4 Division of Dermatology, Washington University School of Medicine, St. Louis, Missouri, USA; 5 Department of Internal Medicine, Washington University School of Medicine, St. Louis, Missouri, USA; Massachusetts General Hospital, Boston, Massachusetts, USA

**Keywords:** siderophores, quorum sensing, iron acquisition, secondary metabolism, urinary tract infection, *Escherichia coli*, virulence regulation, metabolic regulation, host-pathogen interactions

## Abstract

Siderophores are secreted ferric ion chelators used to obtain iron in nutrient-limited environmental niches, including human hosts. While all *Escherichia coli* express the enterobactin (Ent) siderophore system, isolates from patients with urinary tract infections additionally express the genetically distinct yersiniabactin (Ybt) siderophore system. To determine whether the Ent and Ybt systems are functionally redundant for iron uptake, we compared the growth of different isogenic siderophore biosynthetic mutants in the presence of transferrin, a human iron-binding protein. We observed that Ybt expression does not compensate for deficient Ent expression following low-density inoculation. Using transcriptional and product analysis, we found this non-redundancy to be attributable to a density-dependent transcriptional stimulation cycle in which Ybt functions as an autoinducer. These results distinguish the Ybt system as a combined quorum-sensing and siderophore system. These functions may reflect Ybt as a public good within bacterial communities or as an adaptation to confined, subcellular compartments in infected hosts. This combined functionality may contribute to the extraintestinal pathogenic potential of *E. coli* and related *Enterobacterales*.

**IMPORTANCE:**

Patients with urinary tract infections are often infected with *Escherichia coli* strains carrying adaptations that increase their pathogenic potential. One of these adaptations is the accumulation of multiple siderophore systems, which scavenge iron for nutritional use. While iron uptake is important for bacterial growth, the increased metabolic costs of siderophore production could diminish bacterial fitness during infections. In a siderophore-dependent growth condition, we show that the virulence-associated yersiniabactin siderophore system in uropathogenic *E. coli* is not redundant with the ubiquitous *E. coli* enterobactin system. This arises not from differences in iron-scavenging activity but because yersiniabactin is preferentially expressed during bacterial crowding, leaving bacteria dependent upon enterobactin for growth at low cell density. Notably, this regulatory mode arises because yersiniabactin stimulates its own expression, acting as an autoinducer in a previously unappreciated quorum-sensing system. This unexpected result connects quorum-sensing with pathogenic potential in *E. coli* and related *Enterobacterales*.

## INTRODUCTION

Urinary tract infections (UTIs) are one of the most common bacterial infections in clinics and hospitals, with over 7 million doctor visits and 1 million emergency room visits in the United States each year (1). *Escherichia coli* account for ~85% of these infections and are a leading cause of recurrent UTIs (2, 3). As clinical *E. coli* isolates become increasingly resistant to first-line antibiotics, there is renewed interest in better understanding features of these and related *Enterobacterales* that increase their pathogenic potential in human hosts (4).

Among the most prominent host-pathogen interactions in UTIs is the interplay between human factors that limit nutrient iron availability and the corresponding bacterial responses to iron scarcity (5, 6). Normal physiological iron excretion in humans is low, with most iron in the host bound to heme (7, 8). In circulation and tissue, labile iron ions are sequestered by binding to transferrin, a circulating iron transport protein that binds ferric ions in two iron-binding pockets with *K*_*a*_ values of 4.7 × 10^20^ and 2.4 × 10^19^ M^−1^ (9). After the arrival of infecting bacteria, local innate immune responses in tissue intensify this iron sequestration response by introducing additional iron-binding proteins. Epithelial cells and neutrophils introduce lipocalin-2/siderocalin, which sequesters iron in complexes with catechol metabolites (10–12). Lactoferrin, a member of the transferrin family excreted by neutrophils, can bind iron 300 times more tightly than transferrin (13) and, like lipocalin-2, becomes detectible in the urine of patients with UTIs (14). After bacteria establish an early infection, these innate immune responses intensify the baseline iron restriction state maintained by transferrin.

Upon entering the host, infecting *E. coli* counteract host-mediated iron ion sequestration by secreting specialized iron chelators called siderophores, which have been detected in urine from UTI patients (11, 15). Siderophores are small molecules with affinities for iron(III) that make them competitive with mammalian iron-binding proteins (16). Iron(III)-siderophore complexes are avidly imported by dedicated bacterial import systems to support nutritional demands for this critical, growth-limiting nutrient. Intracellular iron released from siderophore complexes then forms an iron-sulfur complex on the ferric uptake regulator (Fur) protein (17, 18), activating feedback repression of siderophore biosynthetic genes. The stereotypical, 19-base sequence motif (the Fur box) involved in this regulatory arrangement is a common criterion for identifying siderophore operons in genomic data (19).

*E. coli*, as well as many other *Enterobacterales* species, secrete the catechol-based siderophore enterobactin (Ent), a siderophore with extraordinarily high iron(III) affinity (*K*_*a*_ ~10^52^) (20). Nevertheless, *E. coli* isolates from patients with UTI encode up to three additional, genetically non-conserved siderophore systems that import siderophores yersiniabactin (Ybt), salmochelin, and aerobactin, respectively. Ybt is the non-Ent siderophore most frequently encountered in clinical urinary isolates (10, 21–23), where it is associated with increased pathogenic potential (4, 24, 25). Why *E. coli* strains with elevated pathogenic potential incur the additional metabolic cost of synthesizing additional siderophores is a longstanding question. One rationale is that these non-conserved siderophores more effectively evade innate immune responses than the Ent system. Another rationale, independent of iron acquisition, is that non-conserved siderophore systems may benefit infection-associated *E. coli* strains through interactions with non-iron transition metal ions, including copper (23) and nickel (22). Given that a single siderophore may perform multiple functions, these rationales are not mutually exclusive.

In the present study, we ask whether Ybt is functionally redundant with Ent for iron uptake. In these experiments, we compared the growth of model uropathogenic *E. coli* (UPEC) strain UTI89 mutants expressing only Ent or Ybt in the presence of human transferrin (hTf). We also chemically complemented cultures of siderophore-deficient strains with purified Ent or Ybt. We used transcriptional reporter constructs for siderophore biosynthetic genes to identify a non-canonical mode of regulation consistent with the characteristics of quorum-sensing (QS) systems. This regulatory mode is retained in the absence of transferrin and during growth in human urine. The results are consistent with a multifunctional role for Ybt that includes autoinduction in a QS regulation in addition to transition metal ion binding.

## RESULTS

### Siderophore-dependent UPEC growth in hTf-containing medium

To determine whether the iron acquisition functions of UPEC siderophore systems are functionally redundant, we first sought to establish a culture condition in which growth is dependent upon siderophore production. The model UPEC strain UTI89 can secrete catecholate siderophores (Ent and salmochelin) and a phenolate siderophore Ybt (24). Ent produced by UTI89 may be secreted directly or modified by C-glucosylation machinery encoded by the *iroA* cassette to form salmochelin and other modified Ents. To identify a siderophore-dependent growth condition, we compared growth of UTI89 with its isogenic, siderophore-deficient mutant UTI89*ΔentBΔybtS* (24). In M63/nicotinic acid/0.2% glycerol medium, which has been previously demonstrated to stimulate UPEC siderophore production (22, 23, 26), UTI89*ΔentBΔybtS* exhibited only a mild growth defect in the late stationary phase relative to UTI89 (see Fig. S1a in the supplemental material). In contrast, hTf addition, previously used to create a siderophore-dependent growth condition (27), led to a marked, highly significant UTI89*ΔentBΔybtS* growth defect relative to UTI89 (*P* < 0.0001 after 9 hours) (Fig. S1b). This growth defect was not observed when hTf was replaced with heat-denatured hTf or an equivalent molar amount of bovine serum albumin (Fig. S1c and d), consistent with a determinative role for intact hTf iron-binding domains. FeCl_3_ supplementation (10 µM) also abolished the hTf-associated UTI89*ΔentBΔybtS* growth defect, consistent with an iron-dependent phenotype (Fig. S1e). Finally, the substitution of hTf with 150 µM of the iron(III) chelator compound, 2,2′-dipyridyl replicated the UTI89*ΔentBΔybtS* (24) growth defect observed with hTf (*P* < 0.01 after 10 hours) (Fig. S1f), which is also consistent with an iron-dependent phenotype. Together, these data are consistent with M63/nicotinic acid/0.2% glycerol/hTf (hereafter abbreviated as M63 + hTf) as a siderophore-dependent growth condition.

### Siderophore system contributions to siderophore-dependent growth

**Fig 1:**
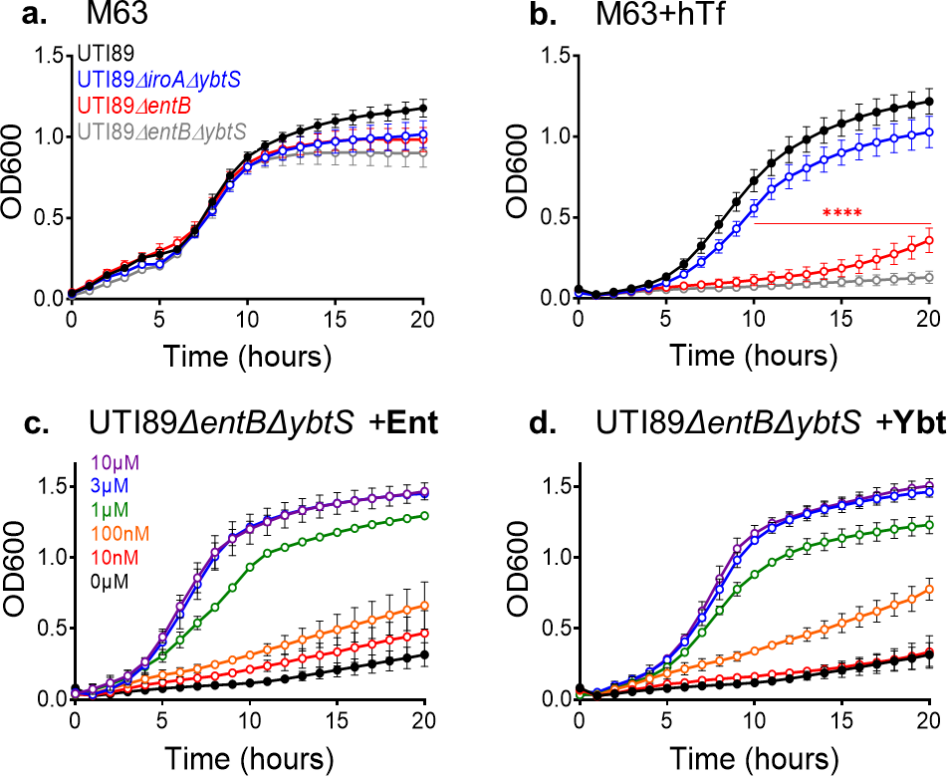
Ybt biosynthesis does not compensate for loss of Ent biosynthesis during siderophore-dependent growth of *E. coli* UTI89 and isogenic mutants. (**a and b**) Growth curves of UTI89 and isogenic siderophore biosynthetic mutants in M63/glycerol media in the absence (**a**) or presence of 3-µM human *apo*-transferrin (hTf, **b**). Unpaired *t*-test comparison of OD600 for single-gene mutant compared to wild-type UTI89 at each time point: ***P < 0.001. (**c and d**) Growth curves of the complete siderophore biosynthesis-deficient mutant UTI89*ΔentBΔybtS* in M63 + hTf containing increasing concentrations of purified Ent (**c**) or Ybt (d).

To determine whether the Ent and Ybt systems are similarly capable of supporting siderophore-dependent growth, we compared growth between UTI89 mutants expressing Ent and Ybt as the sole siderophores (UTI89*ΔiroAΔybtS* and UTI89*ΔentB*, respectively) (Table 1). In the absence of hTf, both mutants grew similarly to UTI89 (Fig. 1a). In the presence of hTf, UTI89*ΔiroAΔybtS* grew similarly to UTI89, but UTI89*ΔentB* exhibited a profound growth deficiency comparable to the siderophore-deficient strain UTI89*ΔentBΔybtS* (*P* < 0.0001 after 9 hours) (Fig. 1b). Comparable results were obtained with substitution of 2,2′-dipyridyl for hTf (Fig. S2). Together, these results are inconsistent with the hypothesis that the Ybt siderophore system is functionally redundant with Ent system-mediated iron acquisition. The results are consistent with a role for Ent, but not Ybt, in supporting UTI89 growth from low cellular density in the presence of hTf.

**TABLE 1:** Bacterial strains used in this study

Strain	Phenotype	References
UTI89	Clinical isolate of acute cystitis	Chen et al. (16)
UTI89Δ*ybtS*	Ybt-deficient UTI89 deletion mutant	Lv and Henderson (28)
UTI89Δ*ybtSΔiroA*	Ybt-deficient and salmochelin-deficient UTI89 deletion mutant	Henderson et al. (24)
UTI89Δ*entB*	Ent-deficient and salmochelin-deficient UTI89 deletion mutant	Henderson et al. (24)
UTI89Δ*entBΔybtS*	Ent-deficient, Ybt-deficient, and salmochelin-deficient (siderophore-null) UTI89 deletion mutant	Lv and Henderson (28)
UTI89Δ*ybtE*	Ybt-deficient UTI89 deletion mutant	Lv and Henderson (28)
UTI89Δ*fur*	Fur deletion mutant lacking iron-mediated Fur repression	Lv and Henderson (28)
UTI89Δ*ybtA*	*ybtA* deletion mutant lacking HPI-encoded transcription activator	Lv and Henderson (28)
UTI89 *p:entC-mCherry_p:ybtP-GFP*	UTI89 with GFP-*ybtP* and mCherry-*entC* reporter plasmids	This work
UTI89*ΔybtE*_ *p:entC-mCherry_p:ybtP-GFP*	Ybt-deficient UTI89 deletion mutant with GFP-*ybtP* and mCherry-*entC* reporter plasmids	This work
UTI89*ΔentB*_ *p:entC-mCherry_p:ybtP-GFP*	Ent-deficient and salmochelin-deficient UTI89 deletion mutant with GFP-*ybtP* and mCherry-*entC* reporter plasmids	This work
UTI89*ΔentBΔybtS*_*p:entC-mCherry_p:ybtP-GFP*	Ent-deficient, Ybt-deficient, and salmochelin-deficient (siderophore-null) UTI89 deletion mutant with GFP-*ybtP* and mCherry-*entC* reporter plasmids	This work
UTI89*Δfur*_*p:entC-mCherry_p:ybtP-GFP*	Fur deletion mutant lacking iron-mediated Fur repression with GFP-*ybtP* and mCherry-*entC* reporter plasmids	This work
UTI89*ΔybtA*_*p:entC-mCherry_p:ybtP-GFP*	*ybtA* deletion mutant lacking HPI-encoded transcription activator with GFP-*ybtP* and mCherry-*entC* reporter plasmids	This work
JPH518	*E. coli* ST131 strain isolated from a UTI patient	Zou et al. (25)
JPH530	*E. coli* ST131 strain isolated from a UTI patient	Zou et al. (25)
JPH544	*E. coli* ST131 strain isolated from a UTI patient	Zou et al. (25)
JPH557	*E. coli* ST131 strain isolated from a UTI patient	Zou et al. (25)
NU-14	UTI model strain	Ohlemacher et al. (29)
SJH734 (*C. diversus*)	*Citrobacter* strain isolated from a UTI patient	Ohlemacher et al. (29)

### Chemical complementation effects on siderophore-dependent growth

We considered that non-redundancy of the Ybt system may arise from deficient Ybt siderophore activity relative to Ent. To test this hypothesis, we chemically complemented UTI89*ΔentBΔybtS* in M63 + hTf with equimolar quantities of either purified Ent or Ybt. We observed a comparable dose-response relationship for the two siderophores, reaching a maximal response at 3 µM for each (Fig. 1c and d). This result is consistent with Ybt and Ent having equivalent siderophore activity in M63 + hTf. Thus, the growth defect of UTI89*ΔentB* in M63+ hTf (Fig. 1b) is not due to an inability of Ybt to substitute for Ent’s siderophore activity.

### Extracellular siderophore accumulation during siderophore-dependent growth

**Fig 2:**
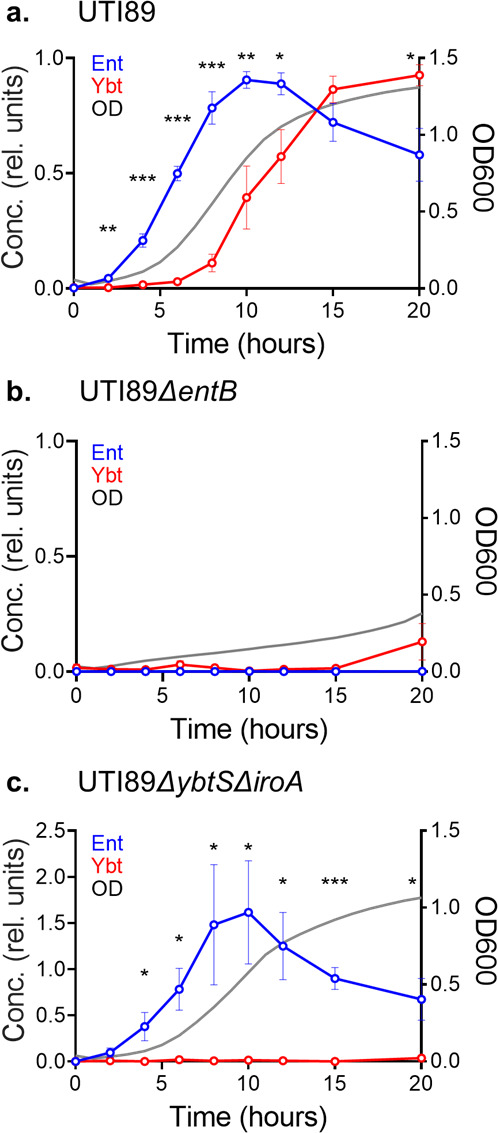
Time course of Ent and Ybt production by *E. coli* UTI89 and isogenic mutants. Liquid chromatography-mass spectrometry was used to quantify Ent (blue) and Ybt (red) content of M63 + hTf medium conditioned by wild-type UTI89 (**a**), UTI89*ΔentB* (b), or UTI89*ΔybtSΔiroA* (c) over 20 hours. Concentration values are relative to maximum values in UTI89. Culture density (optical density at 600 nm) is depicted by the gray plot line. Statistical comparison between relative concentration of Ent and Ybt at each time point: *P < 0.05, **P < 0.01, and ***P < 0.001 by unpaired *t*-test.

We next considered the possibility that Ent and Ybt production differs in our siderophore-dependent culture conditions. To compare siderophore production in M63 + hTf, we used liquid chromatography-mass spectrometry to quantify siderophores in conditioned media over time. In UTI89 cultures, Ybt concentrations exhibited a delayed increase compared to Ent, paralleled the growth (optical density) curve, and were maximal during stationary phase (Fig. 2a). In the Ent-deficient mutant UTI89*ΔentB*, Ybt production kinetics were markedly depressed (Fig. 2b). Conversely, Ent production kinetics were substantially unchanged in the Ybt-deficient mutant UTI89*ΔybtSΔiroA* (Fig. 2c). In the absence of hTf, Ybt production kinetics by UTI89*ΔentB* and Ent production kinetics by UTI89*ΔybtSΔiroA* were both comparable to UTI89 (Fig. S3a to c). These results are consistent with Ybt production during low bacterial cell density culture that is insufficient to overcome the absence of Ent during siderophore-dependent growth.

### Ybt biosynthetic gene transcription is delayed during siderophore-dependent growth

**Fig 3:**
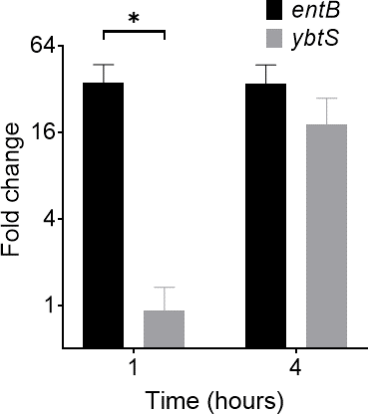
Early siderophore biosynthetic gene transcription in UTI89 assessed by qRT-PCR during siderophore-dependent growth. mRNA fold change of *entB* and *ybtS* biosynthetic gene expression at 1 and 4 hours in M63 media containing 150-µM 2,2′-dipyridyl, normalized to 10-hour time point. *rssA* was used as a housekeeper gene. *P < 0.05 using unpaired *t*-test.

The disparate growth kinetics displayed by UTI89 siderophore mutants may reflect differences in siderophore biosynthetic gene expression during early culture. To test early biosynthetic gene expression, we used real-time quantitative reverse transcription polymerase chain reaction (qRT-PCR) to compare mRNA transcripts from early Ent and Ybt biosynthetic genes (*entB* and *ybtS*, respectively) (Table S1). To accommodate the large media volumes necessary to obtain adequate RNA yield from low-density cultures, we substituted hTf with 2,2′-dipyridyl. At 1 hour of culture, the expression fold change of *entB* was significantly higher than that of *ybtS* (*P* = 0.03). By 4 hours, this difference was diminished and became insignificant (*P* > 0.05) (Fig. 3). This finding is consistent with a UTI89 siderophore system transcriptional response that emphasizes Ent biosynthesis during early growth and Ybt biosynthesis later in culture when cell density is greater.

### Ybt biosynthetic gene upregulation delay is typical of culture populations

**Fig 4:**
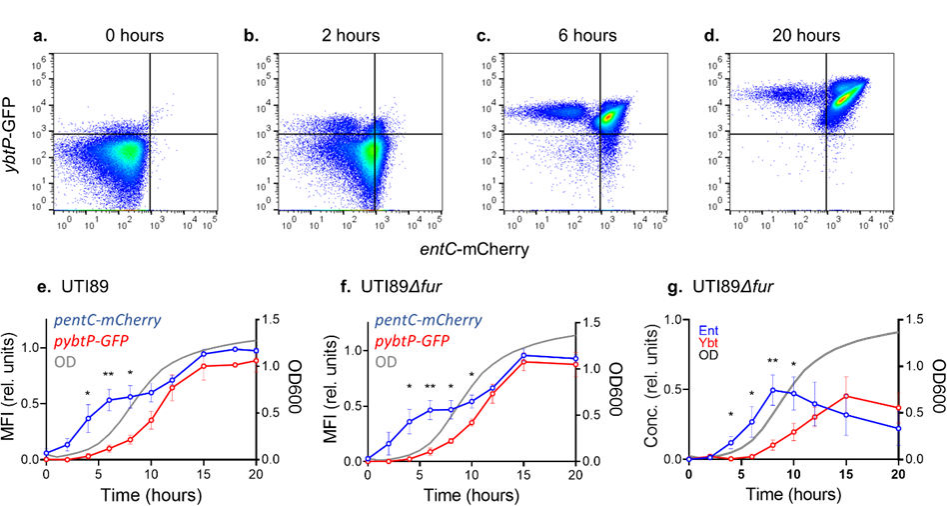
Siderophore biosynthetic operon transcription in UTI89 and its *fur*-deficient mutant assessed by fluorescent reporter expression during siderophore-dependent growth. Fluorescent protein expression in bacterial cultures was measured using flow cytometry. GFP fluorescence controlled by the *Yersinia* operon 1 promoter (*ybtP*-GFP) and mCherry fluorescence controlled by the *entCEBA* promoter (*entC-*mCherry) are displayed. (**a–d**) Representative pseudocolor plots of UTI89 *p:entC-mCherry p:ybtP-GFP* at 0, 2, 6, and 20 hours. Quadrants are gated on non-fluorescent protein cultures. (**e and f**) MFI for each reporter normalized to wild type during growth of UTI89 *p:entC-mCherry p:ybtP-GFP* (**e**) or UTI89*Δfur p:entC-mCherry p:ybtP-GFP* (**f**) in M63 + hTF. (**g**) Ent and Ybt concentrations relative to wild type (UTI89) in M63 + hTf medium conditioned by UTI89*Δfur*. Culture density (optical density at 600 nm) is depicted by the gray plot line. Statistical comparison between relative MFI of *p:entC-mCherry* and *p:ybtP-GFP* at each time point: *P < 0.05, **P < 0.01, and ***P < 0.001 by unpaired *t*-test.

To determine whether Ybt expression was delayed uniformly by cells in culture or is attributable to distinct subpopulations, we created reporter plasmids in which Ent or Ybt biosynthetic operon promoters drive expression of different fluorescent reporters and measured their activity with flow cytometry (Fig. S4a and b) (Table S1). In *p:entC-mCherry*, the *entCEBA* promoter for the main Ent biosynthetic operon controls mCherry expression. In *p:ybtP-GFP*, the *Yersinia* high pathogenicity island (HPI) operon 1 promoter controlling transcription of the first Ybt biosynthetic gene (*ybtS*) controls GFP expression (30) (Fig. 4a through d). In M63 + hTf, mean fluorescence intensity (MFI) (scaled to maximum value) of the *Yersinia* operon 1 reporter in UTI89 *p:entC-mCherry p:ybtP-GFP* was significantly delayed over the first 8 hours compared to the *entCEBA* reporter (2 hours, *P* = 0.054; 4–8 hours, *P* < 0.05; and 10 hours, *P* = 0.076) (Fig. 4e). These results are consistent with the qRT-PCR results and demonstrate a population-wide delay in Ybt biosynthetic gene upregulation by UTI89 in M63 + hTf. Differences in transcriptional activation may relate to the inability of the Ybt system to compensate for the loss of Ent during siderophore-dependent growth.

### Fur repression does not account for delayed Ybt production and expression

We next sought to identify regulatory inputs contributing to differential control of Ent and Ybt biosynthesis. We began with Fur, the canonical siderophore system regulator that represses transcription in the presence of cytosolic iron, although more complex regulatory schemes are possible (31–33). To determine whether differential Fur repression explains the delay in Ybt system transcription, we compared reporter expression and siderophore production between UTI89 and its Fur*-*deficient mutant UTI89*Δfur* (30). We found that UTI89*Δfur* exhibited the same differential *Yersinia* operon 1 reporter stimulation (relative to the *entCEBA* reporter, *P* < 0.05 at 4–10 hours) observed in UTI89 (Fig. 4e and f). UTI89*Δfur* also exhibited the same differential Ybt biosynthetic stimulation (relative to Ent, *P* < 0.05 at 4–10 hours) observed in UTI89 (Fig. 4g). The sustained delay in Ybt biosynthetic upregulation in UTI89*Δfur* is thus consistent with a Fur-independent transcriptional regulatory input on siderophore biosynthesis. Indeed, the comparable transcriptional stimulation observed in UTI89 (relative to UTI89*Δfur*) is consistent with minimal Fur repression in the low iron media conditions used here (minimal medium with hTf).

### Ybt biosynthetic gene upregulation by Ent-null mutant is abolished during siderophore-dependent growth

**Fig 5:**
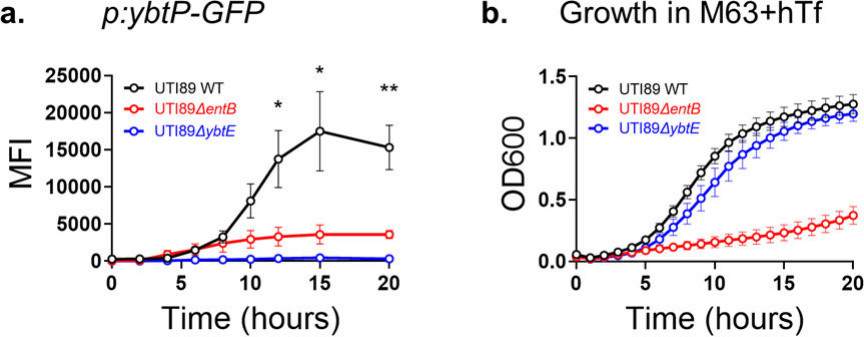
*Yersinia* operon 1 expression and growth of Ent and Ybt biosynthetic mutants in the presence of hTf. UTI89 strains carrying the *p:entC-mCherry* and *p:ybtP-GFP* plasmids were grown in M63 + hTf. *p:ybtP-GFP* reporter expression is displayed as MFI of flow cytometry measurements (**a**) and growth measured by optical density (OD600, **b**). Statistical comparison between wild-type and both mutant strains of the MFI of *p:ybtP-GFP* at each time point: *P < 0.05, **P < 0.01, and ***P < 0.001 by unpaired *t*-test.

To determine whether transcriptional upregulation of Ybt biosynthesis occurs in the UTI89*ΔentB* background, we monitored reporter activity in UTI89*ΔentB p:entC-mCherry p:ybtP-GFP* during growth in M63 + hTf. In this strain, the increase in *Yersinia* operon 1 reporter MFI observed in the wild-type reporter strain was abolished for the duration of the assay (*P* < 0.05, 10–20 hours) (Fig. 5a). This is notable as there is no known direct regulatory connection between the two siderophore systems, and Ent-null UTI89 can grow and produce Ybt comparably to wild-type UTI89 in the absence of competitive iron chelators (Fig. S3) (24). We considered an indirect connection between the two siderophore systems in the M63 + hTf condition. We noted that differences in Ybt biosynthetic transcriptional activity between the wild-type and Ent-deficient reporter strains coincide with differences in culture density (5 hours, *P* = 0.02; 6 hours, *P* = 0.002; and 7–20 hours, *P* < 0.001; Fig. 5b). Unlike the operon 1 reporter, *entCEBA* reporter signal was similar between the wild-type and Ent-deficient strains (0–15 hours, *P* > 0.05) (Fig. S5). This suggested to us the possibility of a density-dependent regulatory input on Ybt biosynthesis.

### Ybt is an autoinducer

**Fig 6:**
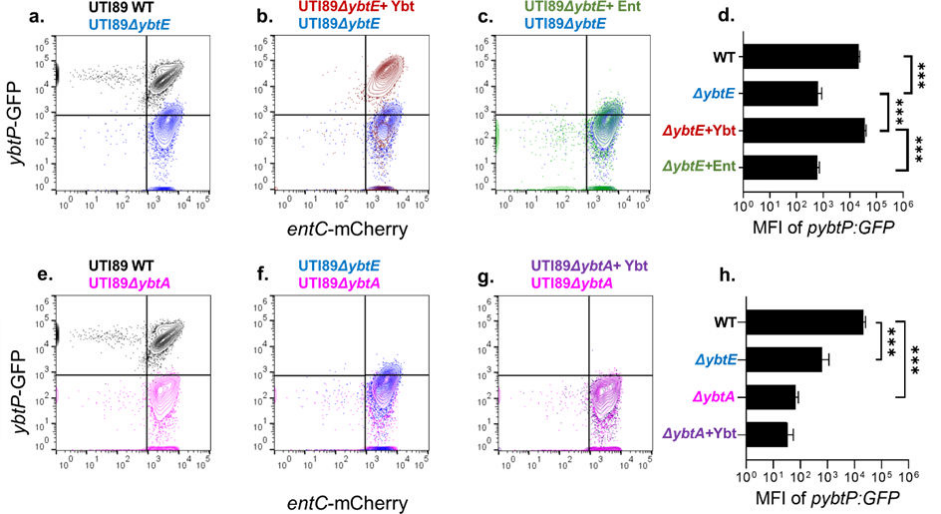
Siderophore biosynthetic operon transcription in UTI89, UTI89 mutants, and with chemically complemented cultures assessed by fluorescent reporter expression. (**a–c and e–g**) Representative pseudocolor plots of strains transfected with *p:entC-mCherry* and *p:ybtP-GFP* after 20 hours of growth in M63 + hTf. Quadrants are gated on non-fluorescent protein cultures. (**d and h**) MFI of *ybtP-*mCherry from each strain at 20 hours. Culture medium was supplemented with 10-nM Ybt (+Ybt, **b, d, g, and h**) or Ent (+Ent, **c and d**). *P < 0.05, **P < 0.01, and ***P < 0.001 by unpaired *t*-test.

We hypothesized that an important Ybt biosynthetic stimulus derives from the density-dependent regulation characteristic of a QS system. A defining feature of QS systems is the production of a secreted molecule that accumulates outside the cell in proportion to population density (34, 35). This secreted molecule—an autoinducer—then upregulates its own production in a concentration-dependent manner, creating a feed-forward autoregulatory loop. Although multiple QS systems have been described in *E. coli* (36–39), we hypothesized that Ybt fulfills the autoinducer role in addition to its siderophore role. To test this, we compared *Yersinia* operon 1 reporter activity between UTI89 *p:entC-mCherry p:ybtP-GFP* and the Ybt biosynthesis-null mutant UTI89*ΔybtE p:entC-mCherry p:ybtP-GFP* during siderophore-dependent growth in M63 + hTf. Despite similar growth curves, the GFP reporter was markedly suppressed in UTI89*ΔybtE* (Fig. 5a). At 20 hours, MFI of *Yersinia* operon 1 reporter was significantly diminished compared to UTI89 wild type (Fig. 6a and d). Chemical complementation of UTI89*ΔybtE p:entC-mCherry p:ybtP-GFP* with purified Ybt restored GFP fluorescence (Fig. 6b and d), while equimolar Ent did not (Fig. 6c and d). Together, these results distinguish Ybt production as subject to autoinductive regulation in which Ybt plays a dual role as effector and signaling molecule. In this context, deficient Ybt accumulation in the medium leads to inadequate Ybt autoinduction at low population density. Ent production, in contrast, is activated independently of population density, facilitates entry into logarithmic growth in M63 + hTf, and facilitates higher Ybt production by the larger bacterial population.

### YbtA is a candidate Ybt sensor

Most QS systems follow a general functional model for density-dependent regulation (40–43). Applying this paradigm to the Ybt system, we anticipated a Ybt-specific response element that increases Ybt biosynthesis. A prominent candidate for this receptor is the AraC-type transcription factor YbtA, which is predicted to possess a ligand-binding domain that may act as a Ybt sensor (18, 30, 44). To assess the role of YbtA in controlling Ybt biosynthesis, we measured reporter activity in the YbtA-deficient mutant UTI89Δ*ybtA.* Compared to UTI89, *Yersinia* operon 1 reporter activity in UTI89Δ*ybtA* remained minimal throughout M63 + hTf cultures (Fig. 6e and h), with a non-significant trend toward lower reporter activity than UTI89Δ*ybtE* at 20 hours (*P* = 0.12) (Fig. 6f and h). Unlike UTI89Δ*ybtE*, the loss of *Yersinia* operon 1 reporter activity in UTI89Δ*ybtA* was not restored by addition of purified Ybt to the culture medium (Fig. 6g and h). Although these results do not definitively identify YbtA as a Ybt receptor, they are consistent with this possible role and show that YbtA is necessary for Ybt biosynthetic gene transcription.

### Differential Ent and Ybt production is typical of multiple clinical urinary isolates

**Fig 7:**
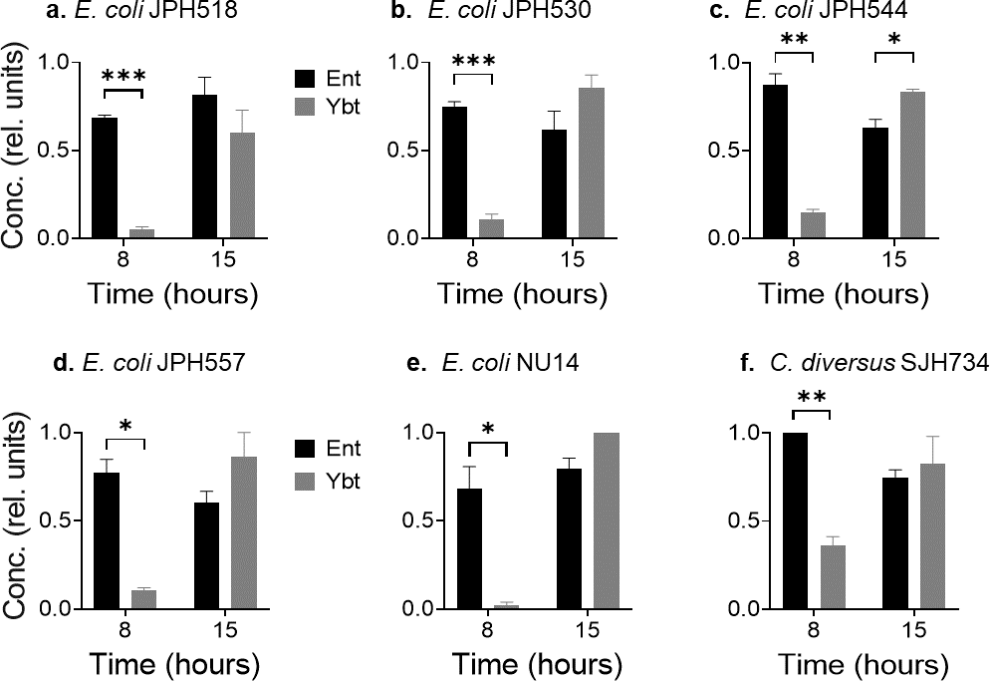
Siderophore production by clinical urinary isolates. Ent and Ybt concentrations relative to UTI89 in M63 + hTf medium. Results at early (8 hour) and late (15 hour) time points are displayed for different *E. coli* (**a–e**) and *C. diversus* (**F**) *P < 0.05, **P < 0.01, and ***P < 0.001 by unpaired *t*-test.

To determine whether other urinary isolates carrying the *Yersinia* HPI exhibit density-dependent Ybt biosynthetic regulation similar to UTI89, we measured siderophore production by genetically distinctive clinical urinary isolates cultured in M63 + hTf (Table 1) (29, 45). In addition to *E. coli*, this included *Citrobacter diversus*, a distinctive *Enterobacterales* species (Fig. 7f). We compared siderophore concentrations of Ent and Ybt during early (8 hours) and late (15 hours) time points. As with UTI89, the Ybt:Ent ratio was higher in late than in early time points for all strains examined (Fig. 7a through f). These findings are consistent with density-dependent Ybt production in multiple urinary *Enterobacterales* isolates, similar to that observed in UTI89.

### Ybt autoinduction in human urine

**Fig 8:**
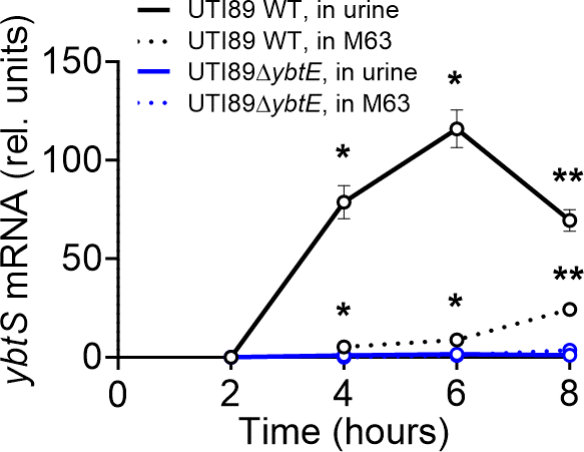
Time-resolved siderophore biosynthetic gene transcription by UTI89 and a Ybt-deficient mutant grown in human urine. UTI89 (black) or UTI89*ΔybtE* (blue) were inoculated into human urine (solid lines) or M63/nicotinic acid/0.2% glycerol medium (dashed line) and harvested for transcriptional analysis at the specified time after inoculation. *ybtS* mRNA (normalized to *gyrA*) is plotted as a function of time. A statistical comparison between normalized *ybtS* mRNA from UTI89 and UTI89*ΔybtE* was performed at each time point: *P < 0.05 and **P < 0.01 by unpaired *t*-test.

To determine whether Ybt autoinduction occurs outside the synthetic medium conditions used above, we monitored *Yersinia* HPI operon 1 transcription (*ybtS*) during growth in filter-sterilized human urine from healthy donors. Using qRT-PCR, we compared *ybtS* mRNA between UTI89 and Ybt-deficient UTI89*ΔybtE*. As incubation time progressed from 2 to 8 hours, *ybtS* mRNA (normalized to *gyrA* mRNA) became significantly greater in UTI89 than in UTI89*ΔybtE*, reaching a maximum difference of 117-fold greater (Fig. 8). In M63/nicotinic acid/0.2% glycerol medium (without hTf), normalized *ybtS* mRNA in an identical inoculum of UTI89 also exceeded that in UTI89*ΔybtE,* reaching a maximum difference 9.6-fold greater. Detectable Ybt had accumulated in both UTI89 culture conditions by the 8-hour endpoint (Fig. S6). Ent accumulated in UTI89 and UTI89*ΔybtE* cultures under both media conditions. These results demonstrate that features of Ybt autoinduction occur in the absence of hTf in both the synthetic M63 medium conditions used above and in a relevant human urinary environment.

## DISCUSSION

**Fig 9:**
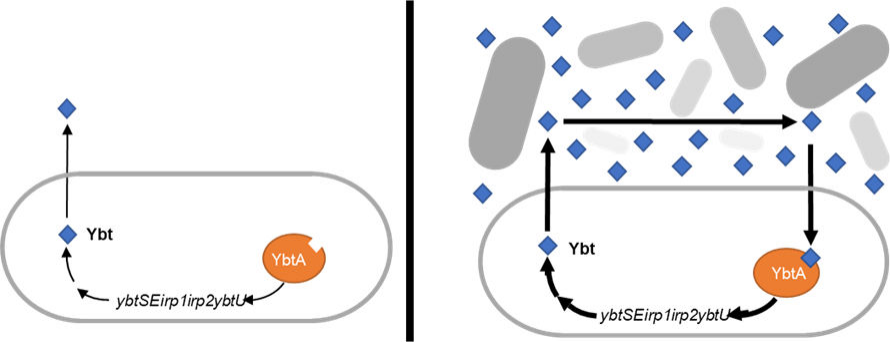
Model of Ybt-mediated QS autoregulation. At low cell density and limited iron availability (*left*), Ybt biosynthesis is insufficient to match the iron uptake activity of the Ent system. Extracellular Ybt accumulates slowly and autoinduction is minimal. With increasing cell density (*right*), extracellular Ybt accumulates sufficiently to bind YbtA and stimulate increased Ybt biosynthesis by each cell. This autoinductive cycle rapidly increases extracellular Ybt as cell density increases.

We find that, during the siderophore-dependent growth of UPEC, the Ybt system is not fully functionally redundant with the genetically conserved Ent system. This is attributable to deficient Ybt biosynthesis at low cell density. Full Ybt biosynthesis, instead, requires a density-dependent transcriptional regulatory cycle in which Ybt acts as an autoinducer (Fig. 9). These characteristics are consistent with the QS regulation, where cellular crowding leads to extracellular accumulation of an autoinductive signal. While QS control of siderophore system activity has been described in other bacteria (40, 41, 46, 47), this is the first description of QS signaling and siderophore activity encoded by the same genetic unit, using the same extracellular small molecule effector.

A combined siderophore and QS system may possess distinctive advantages. Encoding both functions within a single non-conserved, mobile genetic island is genetically efficient, facilitating horizontal gene transfer. In the canonical model involving a freely diffusible siderophore, this arrangement also limits the metabolic cost of Ybt biosynthesis in conditions where Ybt diffuses away and is unlikely to retrieve or control metal ions in the immediate environment. In higher cell density environments, Ybt sharing among producers and recycling of imported Ybt permits more rapid autoinducer accumulation while minimizing biosynthetic costs (23). This view of Ybt as a “public good” contrasts with the Ent system, which *E. coli* appears capable of using as a “private good” at low cell density (48) and requires Ent hydrolysis to release imported iron. Density-dependent siderophore activity may be of particular value in iron-limited host niches where bacterial crowding or confinement is typical. These environments may include epithelial surfaces, biofilms, and intracellular compartments. Ybt autoinduction in human urinary conditions, as observed here (Fig. 8), is consistent with this possibility. Also consistent with this scenario is the previous finding that *ybtS* is the most upregulated UTI89 gene in biofilm-like intracellular bacterial communities residing within bladder epithelial cells during experimental murine UTI (49).

QS stimulation may also optimize Ybt interactions with non-iron transition metal ions such as copper. This may be advantageous during bacterial compartmentalization in the phagolysosome where macrophage-like cells intoxicate bacteria with copper, making Ybt chelation especially advantageous. UPEC compartmentalization may thus serve as an environmental cue, engaging the Ybt autoinductive response and maximizing intraphagosomal copper-Ybt (Cu-Ybt) complex formation. In addition to controlling copper disposition, Cu-Ybt catalyzes superoxide dismutation similar to superoxide dismutases (50). Cu-Ybt has been recently described to stimulate Ybt biosynthesis (30), possibly through YbtA-dependent Cu-Ybt stimulation, augmented autoinduction, and/or signaling by copper ions released from the complex (23, 26). Whether the Ybt system persists among UPEC due to its iron-scavenging activity, its interactions with non-iron metal ions, or both, remains unclear.

QS system regulation of siderophore systems has been previously described in other bacterial orders, where the autoinducer and the siderophore are different molecules. In the prototypical QS bacterium *Vibrio harveyi*, siderophore production is regulated by the canonical Lux QS system (46), which, unlike the Ybt system, represses siderophore expression with increasing cell density. In *Pseudomonas*, the related LasR QS system increases the production of the siderophore pyoverdine with increasing cell density (47). Both the Lux and Las systems use acylhomoserine lactones as the autoinducer. These varying responses to cellular density may reflect adaptation to different environmental stresses and possibly coordination with different, alternative iron acquisition strategies. Nevertheless, the chemical and regulatory diversity of siderophore systems suggests that bacteria have evolved many strategies to control, acquire, and use transition metal ions in their environments.

The *Yersinia* HPI, which is more complex than many siderophore systems, encodes components that are typical of QS system, specifically autoinducer biosynthesis, autoinducer transport, and intracellular receptor/regulator functions (51). The precise functions of these components and their interactions with non-HPI components are incompletely understood. Previous studies have confirmed that Ybt is internalized by an outer membrane transporter (FyuA) (23, 52), suggesting the existence of an intracellular receptor. The most likely candidate for this is YbtA, an AraC-type transcription factor with a well-conserved DNA-binding domain, which is encoded as an independent operon (operon 2) in the HPI and controls transcription of multiple HPI operons (44, 53, 54). Consistent with the autoregulatory function of a QS system, YbtA is predicted to possess an N-terminal ligand-binding domain typical of AraC family proteins that is (18, 30, 44). We speculate that binding to this domain of Ybt, or a derivative thereof, increases transcription of HPI operons and increases Ybt biosynthesis. Further investigation is necessary to test this hypothesis and to construct a more detailed model for its function in *E. coli* and related *Enterobacterales*, which will help us to better understand the host factors exerting selective pressure on these bacteria.

## MATERIALS AND METHODS

### Bacterial strains and culture conditions

Bacterial strains, including UTI89 and previously characterized deletion mutants, used in this study are listed in Table 1. For vector transformations, starter cultures were grown on Luria-Bertani (LB) agar with antibiotics as appropriate overnight at 37°C. Ampicillin (100 µg/mL; Gold Biotechnology), chloramphenicol (34 µg/mL; Gold Biotechnology), and/or kanamycin (100 µg/mL; Gold Biotechnology) were used for selection.

### Bacterial growth curves

Bacteria cultures (3-mL LB medium) were grown overnight with continuous shaking at 37°C. Cells were collected from 1 mL of culture and resuspended in 3 mL of M63 minimal media (0.5-M potassium phosphate, pH 7.4, 10-g/L (NH_4_)_2_SO_4_, 2-mM MgSO_4_, 0.1-mM CaCl_2_, 0.2% glycerol, and 10-g/mL niacin) (24). The cells were then grown for 4-hours shaking at 37°C. One-milliliter of cells were collected and washed with fresh M63 media. Before adding the cells to the 96-well round bottom plate, the wells were filled with 200-µL fresh M63 media with or without 3 µM of hTf (Sigma-Aldrich #T1147) and incubated at room temperature for 30 minutes. Bacteria were added to the 96-well plate for a starting concentration of 0.01 OD (around 8 million CFU). The plate was then grown shaking at 37°C for 20 hours with hourly A_600_ OD measurements using a Tecan SPARK multimode microplate reader with a modular design including an incubator, shaker, and lid lifter (catalog #30086376).

### Ent extraction

Ent was isolated using a method described in previous publications (10, 55). Fractions containing pure Ent were collected, lyophilized, and resuspended in water. Concentrations of metal-free Ent were determined using the Chrome Azurol S assay (56).

### Ybt extraction

Ybt was isolated using a method described in previous publication (30). Fractions containing pure Ybt were collected, lyophilized, and resuspended in water. Concentrations of metal-free Ybt were determined using the Chrome Azurol S assay (56).

### Liquid chromatography-mass spectrometry

Liquid chromatography-mass spectrometry analyses were conducted with a high performance liquid chromatography (HPLC) equipped AB Sciex 4000 QTRAP with a Turbo V ESI ion source run in positive ion mode (Shimadzu, Kyoto, Japan). The samples were injected into a phenylhexyl column (100 by 2.1 mm, 2.7-µm particle) (Ascentis Express, Supelco, Bellefonte, PA, USA) with a flow rate of 0.4 mL/minute. The following gradient was used: Solvent A [0.1% (vol/vol) formic acid] was held constant at 95% and Solvent B [90% (vol/vol) acetonitrile, 0.1% (vol/vol) formic acid] at 5% for 2 minutes. Solvent B was increased to 65% by 6 minutes and to 98% by 8 minutes. Solvent B was then held constant at 98% until 9 minutes before it was decreased to 5% by 11 minutes. Solvent B was then held constant at 5% for 1 additional minute. The collision energy was set at 37 V.

### qRT-PCR analyses

For experiments in chelated medium, UTI89 was grown in 500 mL of M63 minimal media with 150-µM 2,2′-dipyridyl shaking at 37°C. For time-course experiments conducted with human urine, bacterial cultures were grown overnight in M63 minimal media with continuous shaking at 37°C. Bacteria were subcultured into 3-mL M63 minimal media for 4 hours shaking at 37°C. Cells were collected during the exponential phase and were then washed with phosphase-buffered saline (PBS). Washed cells were resuspended in 5 mL of human urine at a final OD ~0.01. Cell pellets were used for qPCR, and the siderophore concentrations in the supernatant were quantified via liquid chromatography-mass spectrometry. RNA was extracted from bacterial cultures using a Qiagen RNA isolation kit. RNA was converted to cDNA, using a Thermo Fisher thermocycler; qRT-PCR was run on the cDNA. Primers for *ybtS* and *entB*, along with housekeepers *rssA* or *gyrA*, were used to measure siderophore biosynthesis transcription (Table S1).

### Fluorescent reporter constructs

Protein expression vector pMAL-c5Xa (NEB) was used as the backbone for constructing the mCherry reporter. The *malE* gene and the *lacI* promoter were restricted from the vector using SacI and KasI restriction enzymes. The *entCEBA* promoter was amplified with primers GK073-F/GK073-R (Table S1) and inserted into the pMAL-c5Xa backbone. The reporter constructs were transformed into respective strains as indicated. An inducible GFP was made by inserting the *Yersinia* operon 1 promoter sequence into plasmid pFCcGi (Addgene) upon restriction with HindIII and XbaI (NEB). The resulting construct was called p*ybtP:GFP* (30). The reporter constructs were transformed into respective strains as indicated.

### Flow cytometry

The bacterial cells isolated from the bacterial cultures were resuspended in 4% paraformaldehyde PBS to fix the cells. The cells were incubated for 30 minutes at room temperature. After 30 minutes, 1 mL of cold PBS was added to quench the fixative. The cells were resuspended in cold FACS buffer (1% BSA and 0.1% sodium azide in PBS). The cells were then measured on a Cytek Aurora flow cytometer (N9-20006 Rev. B) using violet, blue, yellow-green, and red lasers. SpectroFlo software and quality control beads were used to control and read the data from the flow cytometer. The data were then analyzed using FlowJo software. Negative FACS gates were determined using non-fluorescent protein-containing cultures.

### Human urine

The collection of human urine specimens from healthy donors was approved by the Washington University School of Medicine Human Research Protection Office (IRB ID: 201709131). Urine samples were collected from three healthy donors and pooled together in equal volumes. Samples were immediately filter sterilized (0.22-µm pore, Corning, product number 431118) and frozen at −20°C until used for bacterial culture.

## SUPPLEMENTAL MATERIAL

Supplemental material mbio.00277-23-s0001.docx Supplemental tables and figures.

ASM does not own the copyrights to Supplemental Material that may be linked to, or accessed through, an article. The authors have granted ASM a non-exclusive, world-wide license to publish the Supplemental Material files. Please contact the corresponding author directly for reuse.
