# Supplementary material for: Yersiniabactin is a quorum-sensing autoinducer and siderophore in uropathogenic Escherichia coli
Source: mBio. 2024 Jan 18;15(2):e00277-23. doi: 10.1128/mbio.00277-23 (PMC10865836; doi:10.1128/mbio.00277-23)
Supplement: Supplemental material — Supplemental tables and figures. [file mbio.00277-23-s0001.docx]

**
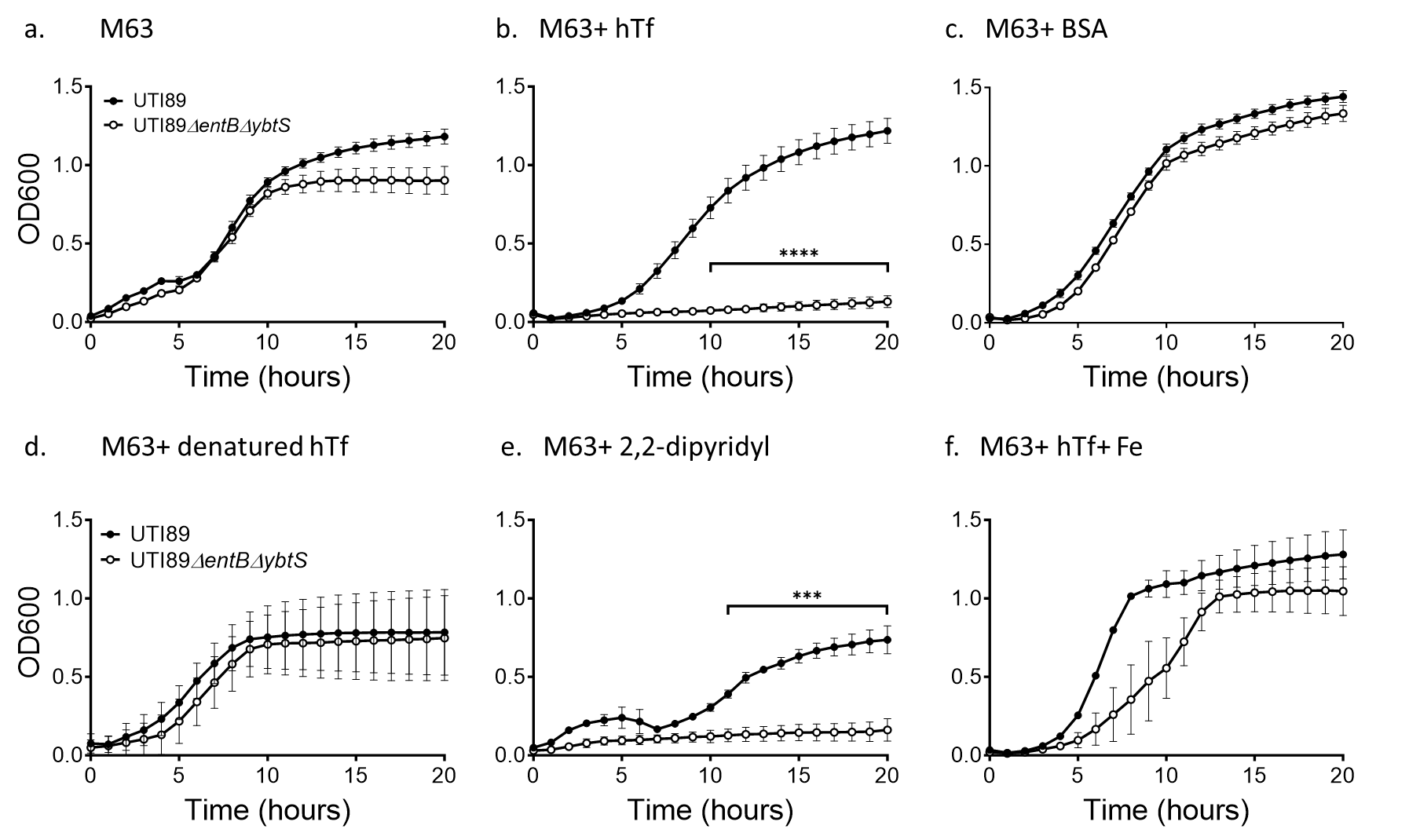
**

**Figure S1: Siderophore-null UTI89 strain does not grow in media supplemented with human transferrin or the iron ion chelator 2,2’-dipyridyl.** Growth curves of wild type UTI89 (black) and siderophore-null UTI89Δ*entBΔybtS* (white) in M63 media alone (a), with 3 μM human Transferrin (hTf) (b), 3 μM bovine serum albumin (BSA)(c), 3 μM heat-denatured hTf (d), 150 μM 2,2’-dipyridyl (e), or 3 μM hTf and 10 μM FeCl_3_(f). Cultures were grown in a 96-well plate shaking for 20 hours at 37 °C. Wells were measured at 600 nm wavelengths every hour by a Tecan plate reader. Unpaired T-test comparison of OD600 for siderophore-null mutant compared to wild type UTI89 at each time point: ***, P < 0.001, ****, P < 0.0001.

**Figure S2: Ybt-only producing strain is diminished in iron-limiting conditions.** Growth curve of UTI89 strains with potential to make Enterobactin (Ent), Salmochelin, and Yersiniabactin (Ybt) (black), Ent only (blue), Ybt only (red), or no siderophores (white). Strains were grown in M63+ 150 μM 2,2’-dipyridyl. Cultures were grown in a 96-well plate shaking for 20 hours at 37 °C. Wells were measured at 600 nm wavelengths every hour by a Tecan plate reader. Unpaired T-test comparison of OD600 for single gene mutant compared to wild type UTI89 at each time point: ***, P < 0.001.

**
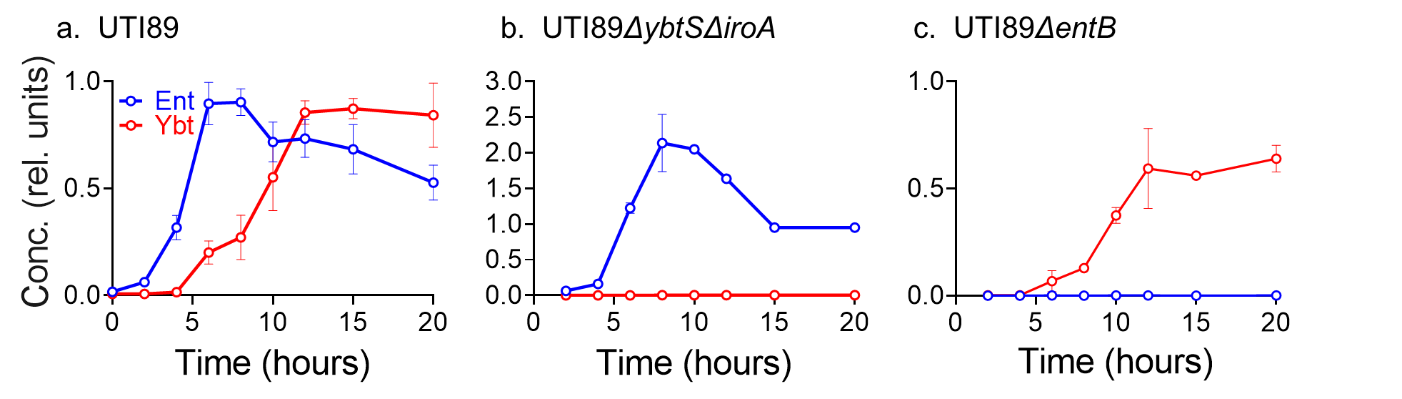
**

**Figure S3:** **Delayed yersiniabactin production by Ent-null UTI89 is sustained in the absence of transferrin.** Siderophore production by UTI89 (a) UTI89*ΔybtSΔiroA* (b), and UTI89*ΔentB* (c) in M63 medium over 20 hours of incubation. Supernatant was sampled at different times, and siderophore concentration in the conditioned medium measured by LC-MS. For each plot, the area ratio for Ent (blue) or Ybt (red) are expressed after normalization to the maximum values achieved by UTI89 for each siderophore.

**a.** *p:entC-mCherry* **b.** *p:ybtP-GFP*

**Figure S4: Maps of reporter plasmids containing fluorescent proteins linked to promoter sequences of operons containing early siderophore biosynthesis genes.**

**
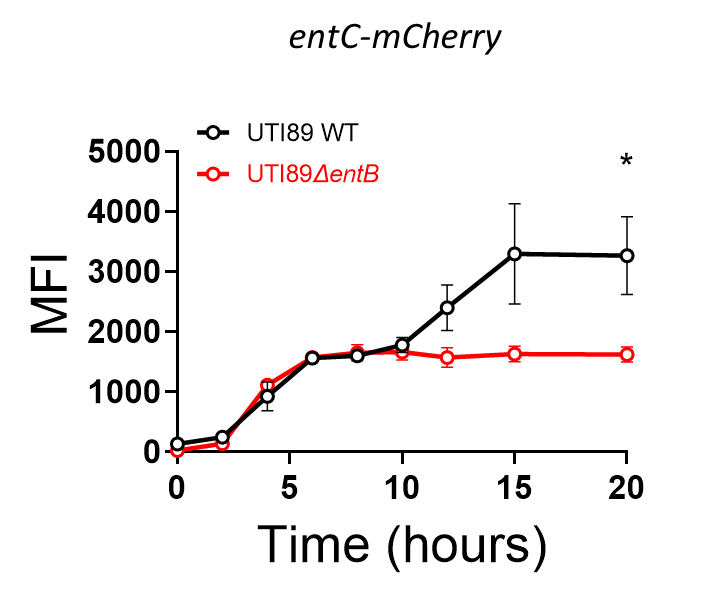
**

**Figure S5: Early *entCEBA* activation is sustained in the Ent-deficient UTI89*ΔentB* mutant.** UTI89 and UTI89*ΔentB* mutants transfected with the *p:entC-mCherry* and *p:ybtP-GFP* plasmids were grown in M63+hTf and expression of fluorescent proteins were measured using flow cytometry. MFI of Ent operon reporter UTI89 WT (black), UTI89*ΔentB* (red). Unpaired T-test used as the statistical test at each time point: *, P < 0.05.


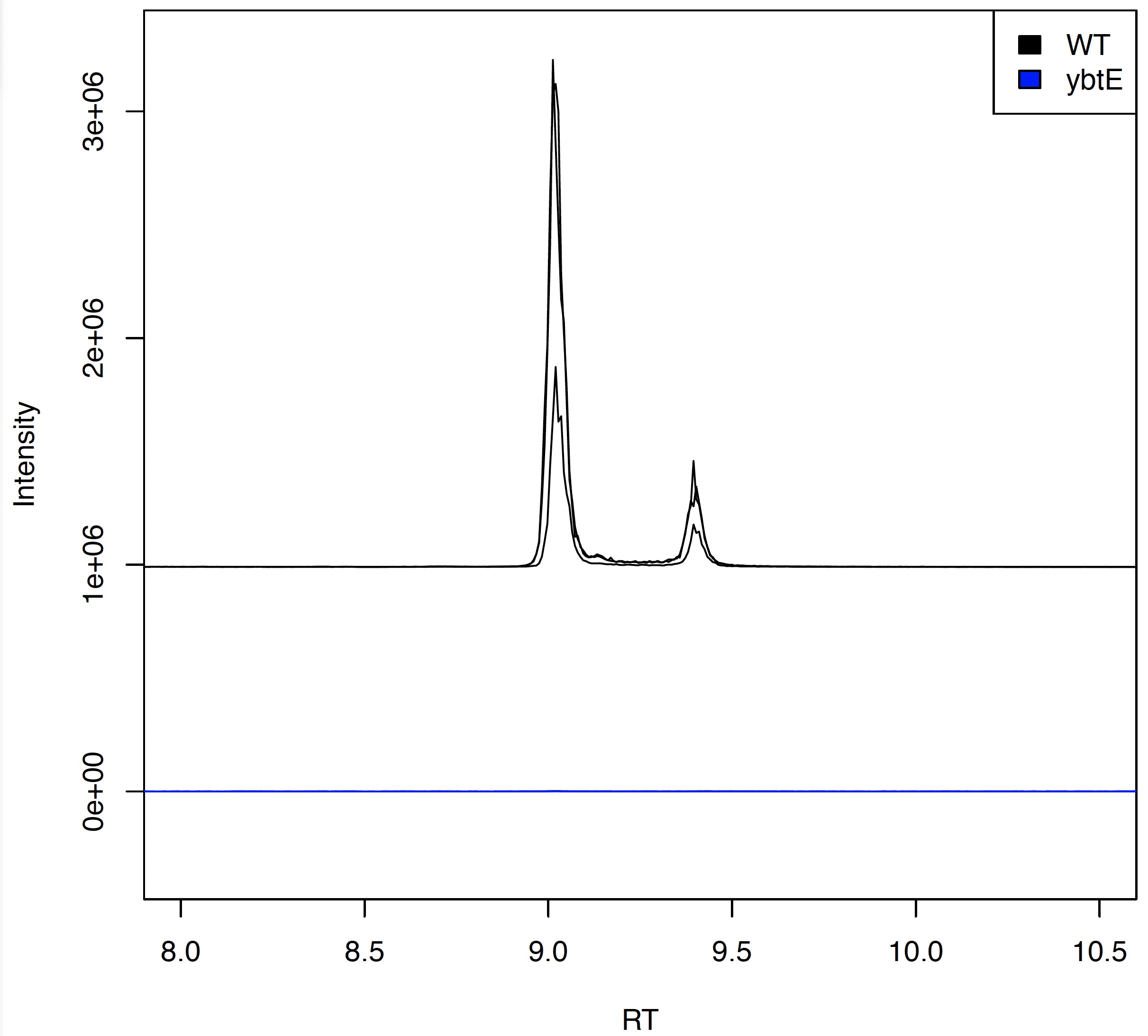

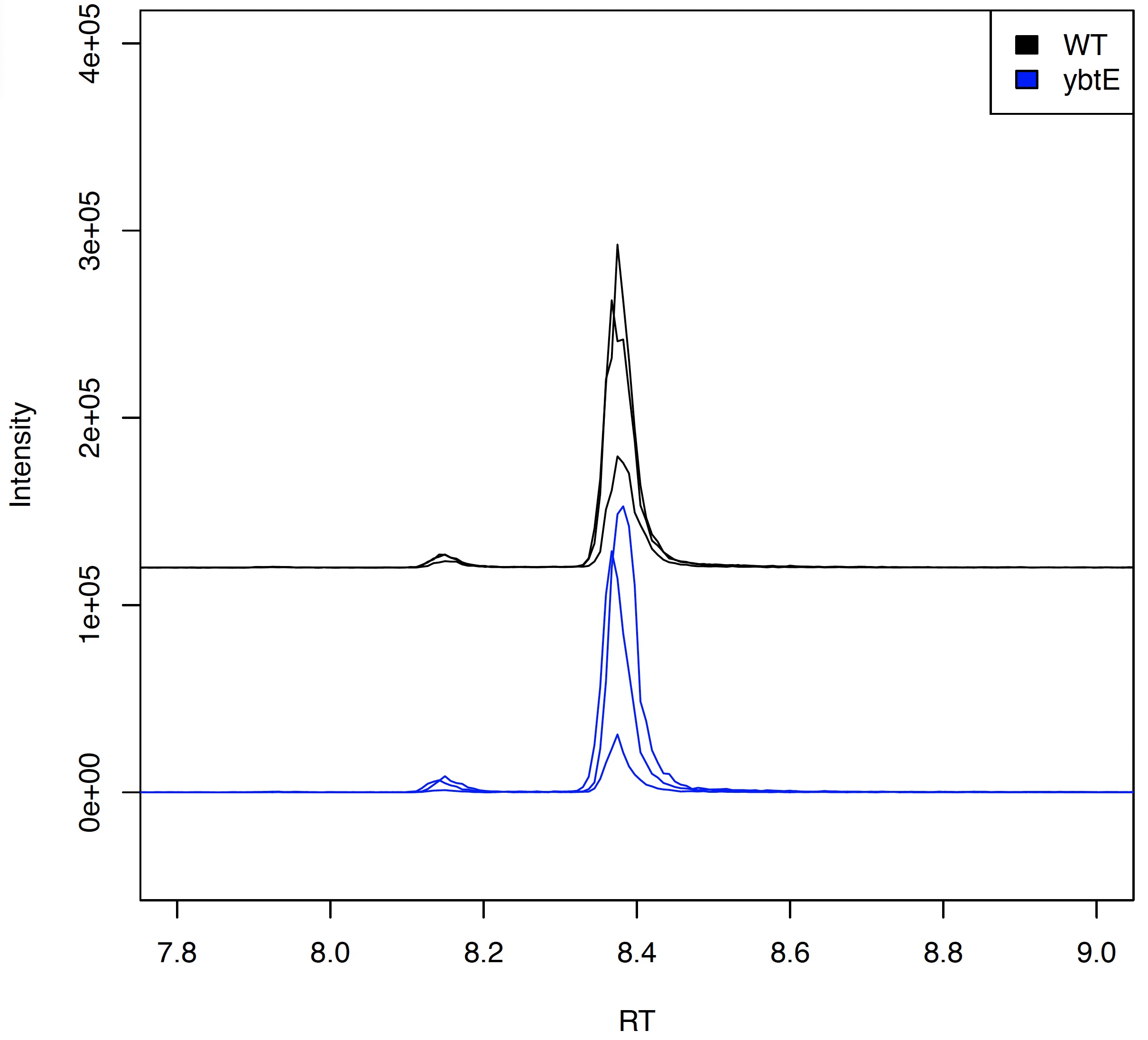

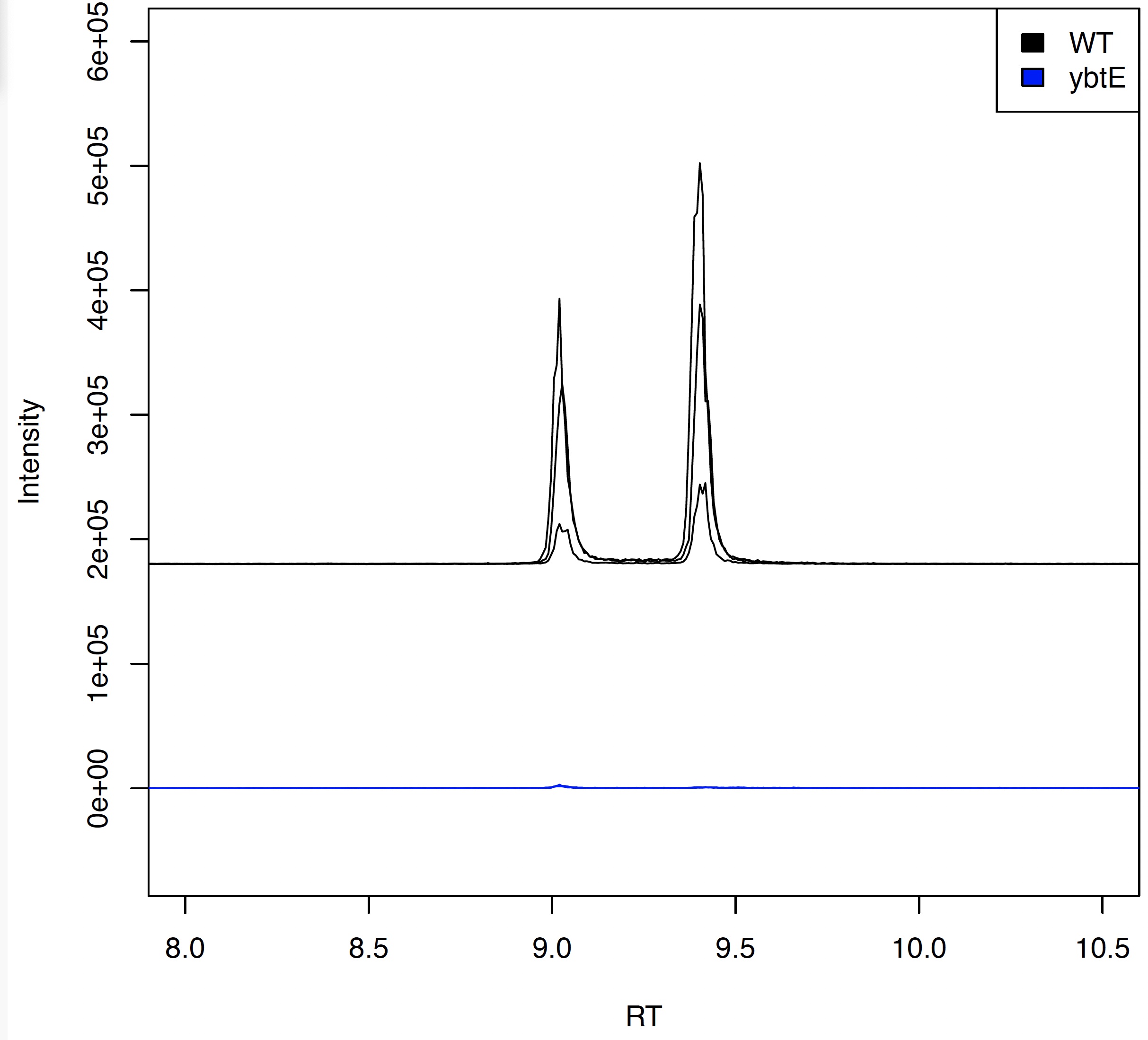

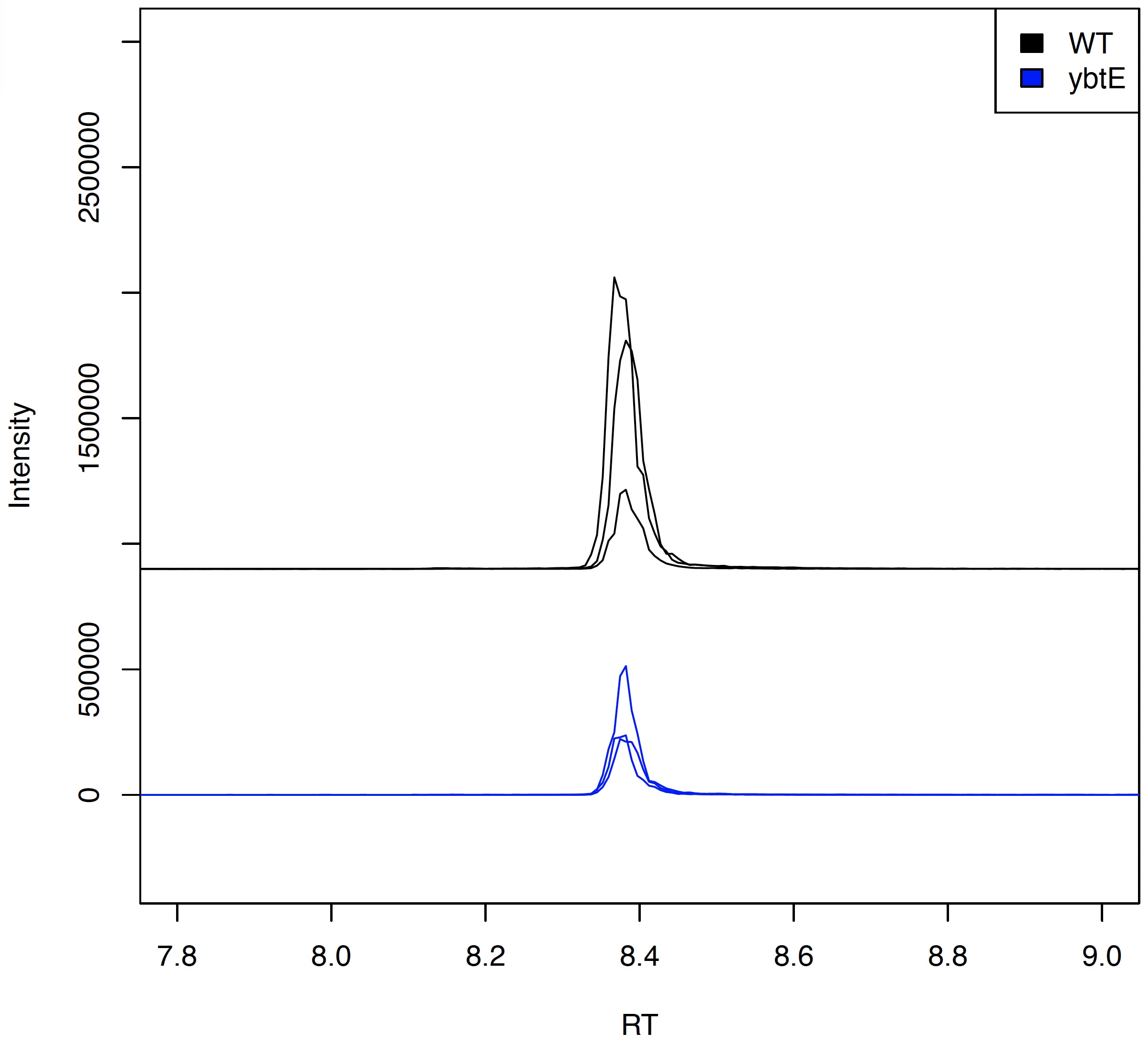


a. Ybt, urine

c. Ybt, M63/nicotinic acid/glycerol

d. Ent, M63/nicotinic acid/glycerol

b. Ent, urine

**Figure S6.** Selected reaction monitoring (SRM) chromatograms for Ybt (a,c) and Ent (b,d) in urine (a,b) or M63/nicotinic acid/glycerol (c,d) supernatants after 8 hour culture with UTI89 (black trace) or UTI89*ΔybtE* (blue trace). The bacteria from these specimens were collected for qRT-PCR analysis (see **Fig 8**). The two peaks in the Ybt-specific chromatograms (a,c) correspond to the two Ybt isomers. The absence of these peaks in the UTI89*ΔybtE* chromatograms confirms the previously established Ybt deficiency of this mutant. The single peaks on the right correspond to cyclic enterobactin, the biosynthetic pathway of which is undisturbed active in both UTI89 and UTI89*ΔybtE.*

**Table S1: Primer sequences.**

| Primer ID | Sequence | Purpose |
| --- | --- | --- |
| *entB* bFwd | ACGTGCCGCGTTGTTAATC | Specific forward primer for *entB* |
| *entB* bRev | TCCACCACCTTTTGCTGTTC | Specific reverse primer for *entB* |
| *ybtS* mFwd | CCTCTTTCGCCTTATTATGCTC | Specific forward primer for *ybtS* |
| *ybtS* mRev | CGCTCGTTTATGTTCCGTC | Specific reverse primer for *ybtS* |
| *rssA* Fwd | ATGCGCGTCTGAAAGAAAGG | Specific forward primer for *rss* |
| *rssA* Rev | CGCGATGGAAATCAAGCGTA | Specific reverse primer for *rss* |
| *gyrA* Fwd | GTAAATACCATCCCCATGGTA | Specific forward primer for *gyrA* |
| *gyrA* Rev | ATTTCCGTATAACGCATTGCC | Specific reverse primer for *gyrA* |
| GK073-F | GATCGGATCCAAGGGCGAGGAGGATAAC | Amplifying entC promoter |
| GK073-R | CTAGAAGCTTTTACTTGTACAGCTCGTCCATG | Amplifying entC promoter |
| GK074-F | CTAGGGATCCCATGGGAGTAACTGAATTTCC | Amplifying operon 1 promoter. |
| GK074-R | GATCGAGCTCCATGACCTGGTTATCTCCCT | Amplifying operon 1 promoter. |
